# Transmitting Pulse Encoding for Beyond-PRT Retransmitting Deception Jamming Detection in Spaceborne Synthetic Aperture Radar (SAR)

**DOI:** 10.3390/s18051666

**Published:** 2018-05-22

**Authors:** Ruijia Wang, Bing Sun, Xing Wang, Siyi Cheng

**Affiliations:** 1 Aeronautics Engineering College, Air Force Engineering University, Xi’an 710038, China; wangxing@afeu.edu.cn (X.W.); csy@afeu.edu.cn (S.C.); 2 School of Electronics and Information Engineering, Beihang University, Beijing 100084, China; arthdaka@buaa.edu.cn

**Keywords:** synthetic aperture radar (SAR), retransmitting deception jamming (RDJ), jamming detection, transmitting pulse encoding

## Abstract

Retransmitting deception jamming (RDJ) degrades and misleads the Synthetic Aperture Radar (SAR) image interpretation by forming false targets. The beyond-Pulse Repetition Time (PRT) RDJ enlarges the effective jamming area without constraining the jammer location to reduce the spaceborne SAR working effectiveness. In order to detect the beyond-PRT RDJ and enhance the working efficiency in electronic countermeasure environment, the transmitting pulse encoding method for use in spaceborne SAR is proposed based on the geometry and signal models of beyond-PRT RDJ. Optimum binary codes with maximum number of detection windows are determined by the encoding procedure. The detected area is found to be proportional to the code length and the encoding efficiencies of even and odd codes are analyzed. The simulation results validate the effectiveness of the transmitting pulse encoding method for beyond-PRT RDJ detection in spaceborne SAR.

## 1. Introduction

Spaceborne Synthetic Aperture Radar (SAR) has been widely used in civilian exploration and military surveillance for its all-weather, long time and large-range detection capability [1]. However, the Retransmitting Deception Jamming (RDJ) can significantly degrade the SAR imaging quality and mislead the image interpretation by modulating false target information on the jamming signal [2,3,4,5]. The jammer of traditional RDJ must be located near the nadir of the SAR to cover a large area, because the traditional RDJ uses the current intercept transmitting signal of SAR to contaminate the current echo of protected target. To enlarge the coverage zone of RDJ without constraining the jammer location, the beyond-Pulse Repetition Time (PRT) RDJ was proposed to counter the spaceborne SAR. The beyond-PRT RDJ uses the current intercept transmitting signal to contaminate the subsequent echoes with one or more PRTs time delay. Hence, the jammer location is greater flexible compared with the traditional RDJ [3]. If the beyond-PRT RDJ signal is received by the spaceborne SAR receiver, the satellite will need to refocus on the same scene during the next orbit period. Hence, the beyond-PRT RDJ has negative impact on the working efficiency of spaceborne SAR. As for spaceborne SAR under electronic counter measures environment, the beyond-PRT RDJ detection capability is crucial and necessary.

To date, most of the research and literature has been focused on the unintentional interference detection and suppression [6,7,8]. The typical unintentional interference is narrowband interference (NBI) and wideband interference (WBI) caused by radio broadcast and TV signals which contaminate the SAR image [9,10,11,12]. The amplitude threshold detection was adopted to identify the NBI in the frequency domain where the NBI amplitude will jump at certain range bins [13]. The Kurtosis statistical detection method was proposed to detect NBI in frequency domain using echo data [14]. The negentropy-based statistical test was utilized to detect the WBI in instantaneous spectrum using short-time Fourier transform (STFT) [15]. In essence, the statistical detection methods depend on the non-coherence of interference and echo. However, aforementioned methods are not suitable for RDJ detection for the following reasons. Firstly, the RDJ signal is coherent with echo and the statistical character of echo contaminated with the RDJ signal is barely changed. Secondly, the power of RDJ signal is precisely calculated based on the false target radar cross section (RCS) and is usually smaller than that of the echo. Hence, the amplitude detection is useless for echo with RDJ signal. Thirdly, and most importantly, the unintentional interference is detected and processed only after the echo data is transmitted from satellite to ground center. In contrast to the unintentional interference, the RDJ will make SAR in a near-realtime electronic adversarial environment requiring active jamming detection capability. The RDJ needs to be detected and processed during the transmission and reception of the signal by the satellite to avoid suffering seriously jamming and losing working efficiency.

Multi-channel, Multi-Input and Multi-Output (MIMO) SAR is being increasingly developed. In [16,17,18,19,20,21,22,23], the azimuth multi-channel SAR was proposed for high-resolution and wide-swath. In [20], the sub-band signal synthesizing method for azimuth multi-channel SAR was researched. The multi-channel cancellation method for jamming suppression and the beam-forming method were proposed [22,23]. Based on cross-track MIMO SAR, the beyond-PRT RDJ suppression method was proposed [16]. However, the RDJ detection is not investigated. Besides, the RDJ detection for SAR system partly differs from target detection for radar [24,25,26]. For normal target detection in radar system, the Neyman-Pearson criterion is adopted and the target echoes need to be detected in noise and interference background. In [24], a unifying framework for multi-channel and multidimensional radar in homogenous plus structured interference was built. In [25], an adaptive direction detector was designed in the presence of interference. Compared with target detection, the purpose of RDJ detection is enabling the SAR system to detect the RDJ and perceive the electromagnetic environment.

In this paper, the transmitting pulse encoding method is proposed to detect the beyond-PRT RDJ. Although the beyond-PRT RDJ pulse and target echo pulse are identical, owing to the propagation distance and time delay modulation, the beyond-PRT RDJ signal will lag the target echo signal by some PRTs. By adjusting the transmitting pulse sequence, the beyond-PRT RDJ signal will be detected in the time-domain. In particular, a binary code is utilized to modulate the transmitting pulse sequence, whereby “1” signifies a transmitting pulse and “0” signifies no transmitting pulse. Various codes are researched to detect the beyond-PRT RDJ with arbitrary time delay and with optimum detection efficiency.

This paper is organized as follows: Section 2 presents the spaceborne SAR with beyond-PRT RDJ model and an example of the transmitting pulse encoding method. Section 3 introduces the transmitting pulse encoding method and analyzes the encoding efficiency of odd and even codes. Section 4 presents the results of simulation. Section 5 concludes the paper.

For the convenience of the reader, the following notation is used: *S_J_* represents the beyond-PRT jamming signals. *S_r_* represents the transmitting signal of SAR. *Y* is the echoes with beyond-PRT RDJ signals. *R_J_* is the slant distance between the jammer and the SAR and *R_P_* is the slant distance between the point target and the SAR. *MT*{ } is the time delay operation of signal matrix. *T_c_* represents the transmitting pulse code and *T* is the encoding part of *T_c_*. and *L* is the length of *T*. *N_best_* is the number of optimum code. *N_d_* is the total effective detection window of optimum code.

## 2. Spaceborne SAR Beyond-PRT RDJ Model

### 2.1. Geometry Model

**Figure 1:**
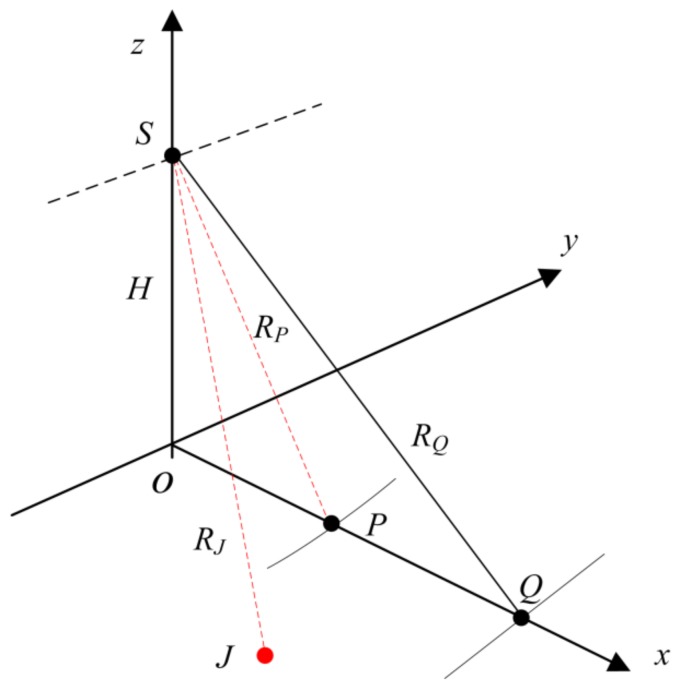
The beyond-PRT RDJ scenario.

An illustration of the beyond-PRT RDJ scenario is provided in Figure 1. The arbitrary protected target *P* is located in front of the jammer *J*. The location of *Q* is the outermost point for the jammer and thus, *R_Q_* represents the largest effective jamming slant range of jammer *J* with constant jamming power. The SAR on the satellite *S* flies along the *y*-axis (as shown by the dotted line). The *R_J_* and *R_P_* represent the slant ranges from the jammer and the protected target to the satellite, respectively.

With traditional RDJ, the jammer must be located closer to the origin *O* than the protected target *P* to effectively conceal the location of *P*. Thus, if the slant range between jammer and satellite is larger than the slant range between protected target and satellite, the traditional RDJ will lose efficacy. However, the beyond-PRT RDJ is utilized to expand the effective coverage of the jammer and so, as Figure 1 shows, the jammer in the beyond-PRT RDJ scenario can be located between *P* and *Q*. Accordingly, the jamming detection method needs to continuously detect the jammer in the range (*R_P_*, *R_Q_*).

### 2.2. Signal Model

The RDJ model is as follows [2,16]:

(1) $$\begin{array}{c} S_{J}\left(t\right)=\sum_{j}\left[S_{r}\left(t\right)*\delta \left(t-\frac{2R_{J}}{c}-\Delta \tau_{dj}\right)\right]\exp\left\{j\varphi_{j}\left(t\right)\right\}\\ t=k\eta +\tau \;0\leq \tau <\eta \end{array}$$

where *S_r_*(*t*) is the transmitting signal, *R_J_* is the single propagation slant range from jammer to satellite, $\Delta \tau_{\mathrm{dj}}$ represents the time delay of the *j*-th false target, $\varphi_{j}(t)$ denotes the *j*-th false target modulating phase. η and τ represent the azimuth and range components of time *t*, respectively. In practice, the linear frequency modulation (LFM) signal is adopted as the transmitting signal. Hence, the baseband signal of RDJ can be expressed as a function of the azimuth and range components as follows:

(2) $$\begin{array}{ll} S_{J}\left(\tau ,\eta \right) & =\omega_{r}\left\{\tau -{\left\lfloor \frac{2R_{J}\left(\eta \right)}{c}+\Delta \tau_{d}\right\rfloor }_{T_{a}}\right\}\omega_{a}^{\prime }\left(\eta -\eta_{J}\right)\\ & \times \exp\left\{j\pi K_{r}{\left(\tau -{\left\lfloor \frac{2R_{J}\left(\eta \right)}{c}+\Delta \tau_{d}\right\rfloor }_{T_{a}}\right)}^{2}\right\}\\ & \times \exp\left\{-j2\pi f_{0}\frac{2R_{J}\left(\eta \right)}{c}\right\}\\ & \times \exp\left\{-j2\pi f_{0}\frac{2\Delta R\left(\eta \right)}{c}\right\} \end{array}$$

where *T_a_* is the PRT, *K_r_* is the frequency modulation rate, the transmitting signal center frequency is *f*_0_, $\omega_{r}\{\cdot \}$ and $\omega_{a}\{\cdot \}$ represent the pulse envelope of range and azimuth components respectively, ⌊·⌋ indicates the modulus operation which presents the time delay of beyond-PRT RDJ and ΔR is the instantaneous difference in slant ranges of jammer and protected target, which represents the phase modulation of beyond-PRT RDJ.

In a scenario given in Figure 1, the jammer needs to conceal the protected target *P*. Accordingly, the time delay and the instantaneous slant range difference or modulation phase must satisfy the following equations:

(3) $$\left\{ \begin{array}{l} \Delta \tau_{d}=\frac{2R_{P}\left(\eta -mT_{a}\right)}{c}+mT_{a}-\frac{2R_{J}\left(\eta \right)}{c}n\\ \Delta R\left(\eta \right)=R_{P}\left(\eta -mT_{a}\right)-R_{J}\left(\eta \right) \end{array}\right.$$

With traditional RDJ, the time delay difference $\Delta \tau_{d}$ must greater than 0 and the *m* is equal to 0. This means that *R_J_* must be less than *R_p_* and thus, the jammer must be located closer to the origin *O* than the protected target. Hence, the protected area is limited by the jammer location. However, for the beyond-PRT RDJ, the *m* can be a non-zero integer to ensure that $\Delta \tau_{d}$ is greater than 0 and *R_J_* can be greater than *R_p_*.

The transmitting signal can be expressed in matrix form as follows:

(4) $$S_{r}={\left[s_{1},\cdots s_{i},\cdots ,s_{N}\right]}^{T}$$

where the vector *s_i_* represents the *i*-th sample of the azimuth component of the signal and there are *N* samples in total. Supposing that each azimuth signal has *M* samples, the azimuth signal *s_i_* is as follows:

(5) $$s_{i}={\left[s_{i}(1),\cdots ,s_{i}(M)\right]}^{T}$$

The beyond-PRT RDJ can be expressed in matrix form as follows:

(6) $$S_{J}=MT{\left\{E⊕S_{r}\right\}}_{m}$$

where *E* is the modulation phase vector as follows:

(7) $$\begin{array}{ll} E & ={\left[\exp\left(j\varphi_{1}\right),\cdots ,\exp\left(j\varphi_{i}\right),\cdots ,\exp\left(j\varphi_{N}\right)\right]}^{T}\\ & ={\left[\exp\left(-j4\pi \frac{\Delta R_{1}}{\lambda_{0}}\right),\cdots ,\exp\left(-j4\pi \frac{\Delta R_{i}}{\lambda_{0}}\right),\cdots ,\exp\left(-j4\pi \frac{\Delta R_{N}}{\lambda_{0}}\right)\right]}^{T} \end{array}$$

where ⊕ is the Hadamard product and *MT*{*A*}*_m_* represents the time delay operation of jammer, whereby each row of matrix *A* is downwards shifted by *m* − 1 rows and the first *m* − 1 rows are replaced with zero vectors. Thus, the time delay operation of jammer is as follows:

(8) $$MT{\left\{A_{NM}\right\}}_{m}=\left[\begin{array}{ccc} 0_{11} & \cdots & 0_{1M}\\ \vdots & \ddots & \vdots \\ 0_{\left(m-1\right)1} & \cdots & 0_{\left(m-1\right)M}\\ a_{11} & \cdots & a_{1M}\\ \vdots & \ddots & \vdots \\ a_{\left(N-m+1\right)1} & \cdots & a_{\left(N-m+1\right)M} \end{array}\right]$$

Hence, the jamming signal *S_J_* is as follows:

(9) $$S_{J}={\left[\begin{array}{cccccc} O_{1} & \cdots & O_{m-1} & s_{1}e^{j\varphi_{1}} & \cdots & s_{N-m+1}e^{j\varphi_{N-m+1}} \end{array}\right]}^{T}$$

The rank of *S_J_*, *R*(*S_J_*), is less than *N*. Ignoring the impact of noise, the echo *Y* with beyond-PRT RDJ is as follows:

(10) $$Y=S_{r}+S_{J}=\left[\begin{array}{c} s_{1}\\ \vdots \\ s_{m-1}\\ s_{m}+s_{1}e^{j\varphi_{1}}\\ \vdots \\ s_{N}+s_{N-m+1}e^{j\varphi_{N-m+1}} \end{array}\right]$$

### 2.3. Example of Transmitting Pulse Encoding

**Figure 2:**
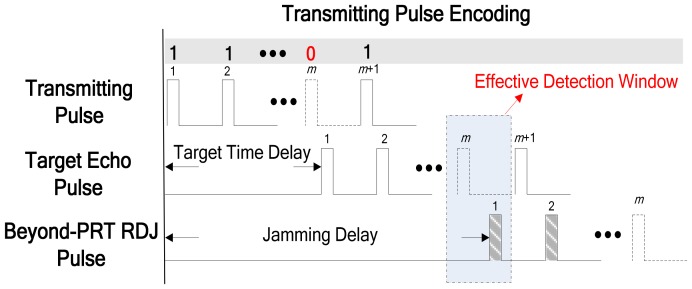
An example of transmitting pulse encoding for beyond-PRT RDJ detection.

As can be seen in Figure 2, the *m*-th transmitting pulse is instead not transmitted as the corresponding element of the binary code is 0 for example. Hence, there will not be any legitimate target echo pulses in the effective detection window. However, owing to the time delay modulation and the propagation delay, the first pulse of beyond-PRT RDJ is received in the effective detection window as shown in Figure 2. Although both the location and time delay modulation of jammer are unknown in practical applications, the slant ranges of the detected area, where the jammer may be placed, can be calculated in advance. For example, the detected area is between *R_P_* and *R_Q_*.

The binary code *T_C_* consists of two parts: the first part, consisting of *L* bits, are used for detection and the second part is normal transmission with *N*−*L* bits of “1”. The code word is expressed as follows:

(11) $$T_{C}={\left[\begin{array}{cc} \underset{L}{\underset{︸}{\begin{array}{ccc} T\left(1\right) & \cdots & T\left(L\right) \end{array}}} & \underset{N-L}{\underset{︸}{\begin{array}{ccc} 1 & \cdots & 1 \end{array}}} \end{array}\right]}^{T}$$

where *T*(*i*) is the *i*-th detection element, which can take on a value of 1 or 0. Hence, the echoes utilized transmitting pulse code are given as follows:

(12) $$Y_{C}={\left[\begin{array}{cc} \underset{L}{\underset{︸}{\begin{array}{ccc} T\left(1\right)s_{1} & \cdots & T\left(L\right)s_{L} \end{array}}} & \underset{N-L}{\underset{︸}{\begin{array}{ccc} s_{L+1} & \cdots & s_{N} \end{array}}} \end{array}\right]}^{T}$$

**Figure 3:**
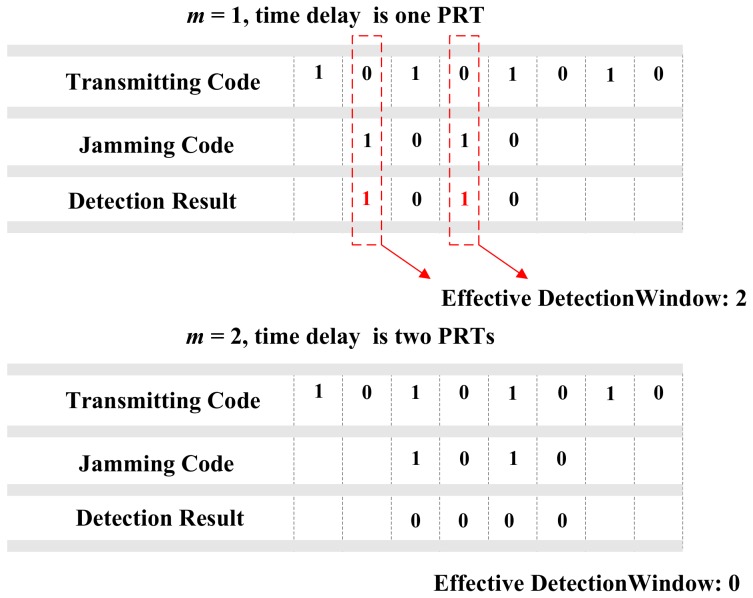
The example of transmitting time sequence encoding detection.

In the above example, we supposed that the transmitting code *T* is [1010]. The beyond-PRT RDJ detection is shown in Figure 3.

As Figure 3 shows, when the time delay of the beyond-PRT RDJ is one PRT, there are two effective detection windows for the supposed transmitting code *T*. When the time delay of beyond-PRT is two PRTs, there are no effective detection windows which mean that the transmitting code *T* may not be effective in RDJ detection for all potential time delays. Hence, the optimum code needs to be determined in different code length.

There are two requirements of the optimum codes. First, there should be at least one effective detection window for each time delay of the beyond-PRT RDJ to ensure the SAR can continuously detect the beyond-PRT RDJ with any potential time delay. Second, the summary of effective detection window number in each time delay is maximum, which make sure the code own the maximum average detection quantity. Moreover, the number of the optimum codes number in different code length is the more the better. Because that the diversity of the optimum code means that the jammer is hard to recognize the time sequence of transmitting pulse.

## 3. Transmitting Pulse Encoding Method and Analysis of Encoding Efficiency

### 3.1. Encoding Method

Based on the two standards for the optimum code, the encoding method is introduced and the optimum codes corresponding different length are got and shown in this section. The binary encoding method is adopted and the code length is determined in proportion with the detected area. So, if the jammer is located within the area defined by slant range interval [*R_N_*, *R_Q_*], where *R_N_* is the nearest slant range of detected area (usually equal to *R_P_*) and *R_Q_* is the maximum slant range of the jammer, the code length can be determined as follows:

(13) $$L=\left\lceil \frac{2\left(R_{Q}-R_{N}\right)}{cT}\right\rceil$$

where ⌈·⌉ symbolizes the ceiling function. Supposing that the pulse must be transmitted in the first azimuth time, this means that *T*(1) is equal to 1. Then, the remaining *L* − 1 bits can form 2^(*L*−1)^ codes, each corresponding to a decimal integer *d* in the range [0, 2^(*L*−1)^-1]. The optimum codes are selected in the procedure detailed as follows:

- *Step 1*: Initialization of transmitting code *T* and jamming code *W_J_*.
  Let *d* = 0 and *T* = *B*(*d*) which converts *d* from decimal to binary. Let *W_J_* = *T*.
- *Step 2*: Right-shift operation on jamming code *W_J_*.

The right-shift number m denoted in (3) represents that the jamming signal lags the target echo signal by *m* PRTs. The maximum number of right-shifts that can be performed is *L* − 1. The right-shift operation is as follows:

(14) $$W_{J}=W_{J}\to 1$$

- *Step 3*: Exclusive OR operation of *W_J_* and *T*.

*T* is periodically extended to match the length of the right-shifted jamming codes *W_J_*. The exclusive OR operation is as follows:

(15) $$W_{E}=W_{J}\;Eor\;T$$

- *Step 4*: Obtaining the beyond-PRT RDJ detection result *W_D_*.

Determine *W_D_* as follows:

(16) $$W_{D}\left(i\right)=\{\begin{array}{l} 0\;W_{E}\left(i\right)-T\left(i\right)\leq 0\\ 1\;W_{E}\left(i\right)-T\left(i\right)=1 \end{array}$$

where 1 represents successful detection of jamming and 0 represents unsuccessful detection of jamming. Thus, the number of 1 element in *W_D_* represents the number of effective detection windows, denoted by *N_s_*.

- *Step 5*: Judging the effectiveness of transmitting code *T*.

If *N_s_* is greater than or equal to 1, it means the current *T* is a valid transmitting code for jamming detection. Then, the current *N_s_* can be stored and the procedure is repeated, starting from Step 2, to obtain other shifted jamming words *W_J_*. If *N_s_* is equal to 0, it means the current *T* is an invalid transmitting code for jamming detection. Then, the procedure is repeated, starting from Step 1 with *d* = *d* + 1.

- *Step 6*: Determining the optimum transmitting pulse codes.

Determine the total number of effective detection windows *N_d_* by summing the effective detection windows *N_s_* for each right-shift of *W_J_*. By comparing the *N_d_* of all transmitting codes *T*, the optimum transmitting codes can be obtained by selecting those codes with the maximum *N_d_* and non-zero *N_s_* for each right-shift of *W_J_*.

**Figure 4:**
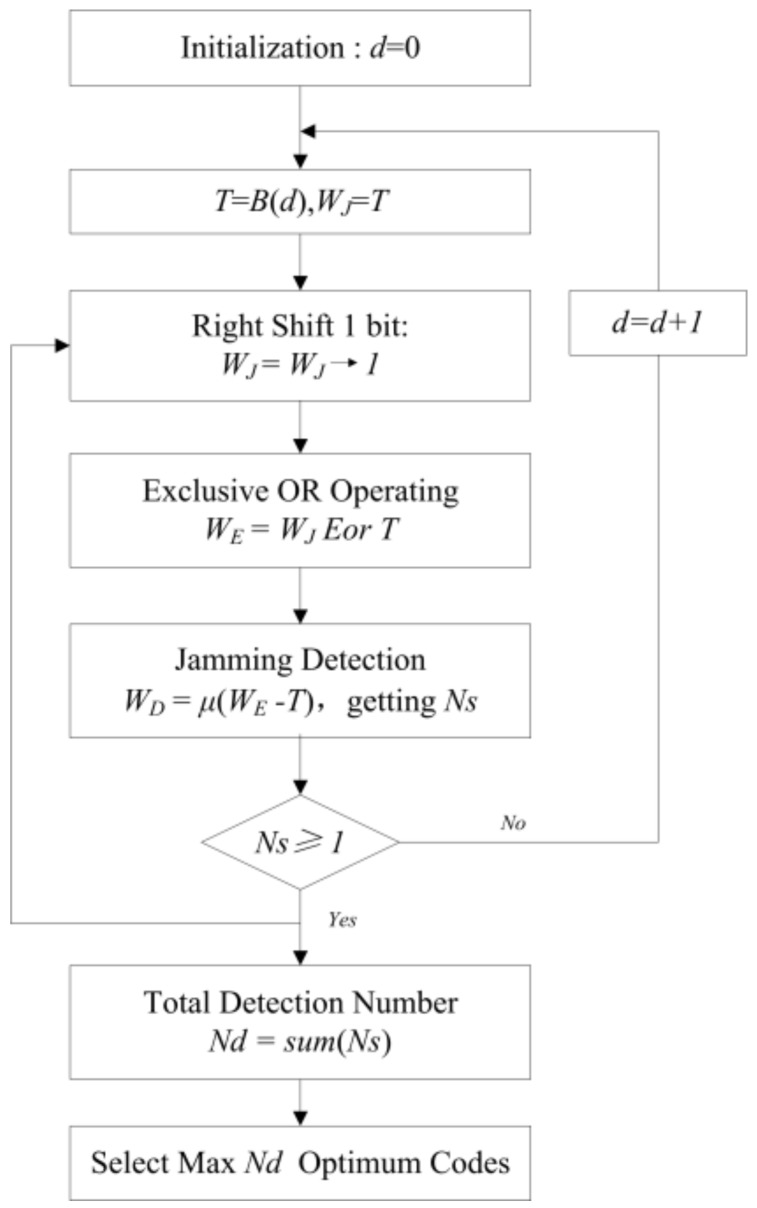
Encoding method flowchart.

In addition, *N_d_* is utilized to evaluate the detection efficiency of transmitting codes *T*. A flowchart of the transmitting pulse encoding method is given in Figure 4.

**Figure 5:**
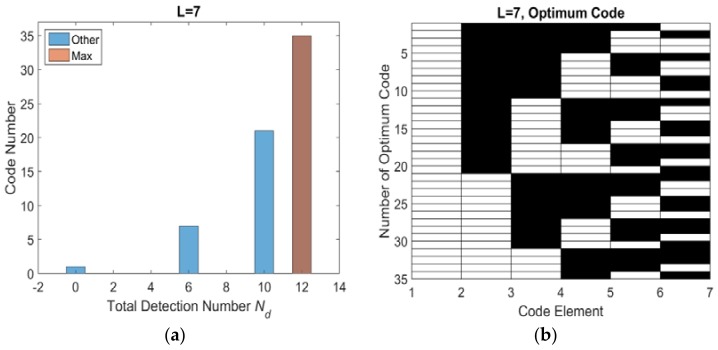
*L* = 7 optimum codes and corresponding *N_d_* (**a**) histogram distribution of *N_d_* (**b**) optimum codes.

**Figure 6:**
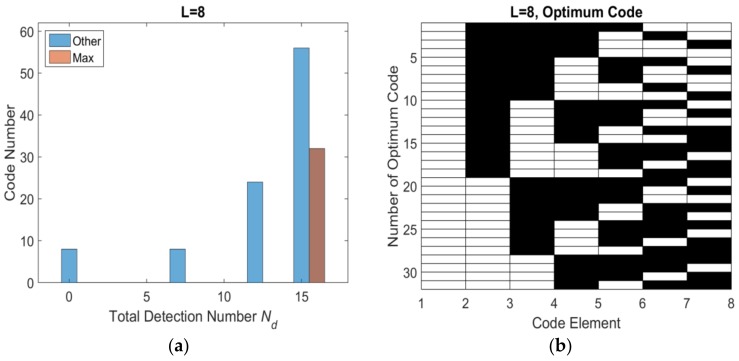
*L* = 8 optimum codes and corresponding *N_d_* (**a**) histogram distribution of *N_d_* (**b**) optimum codes.

**Figure 7:**
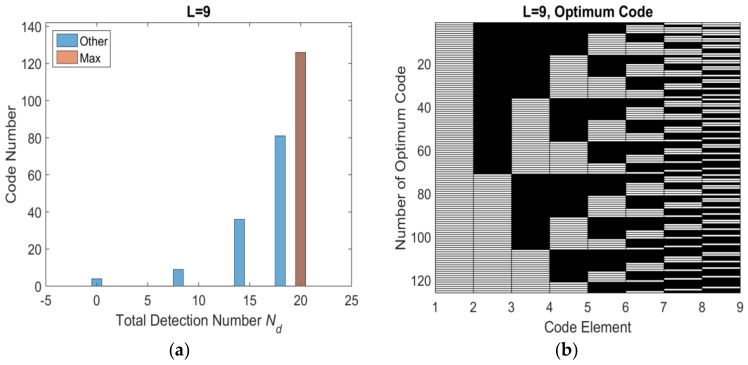
*L* = 9 optimum codes and corresponding *N_d_* (**a**) histogram distribution of *N_d_* (**b**) optimum codes.

**Figure 8:**
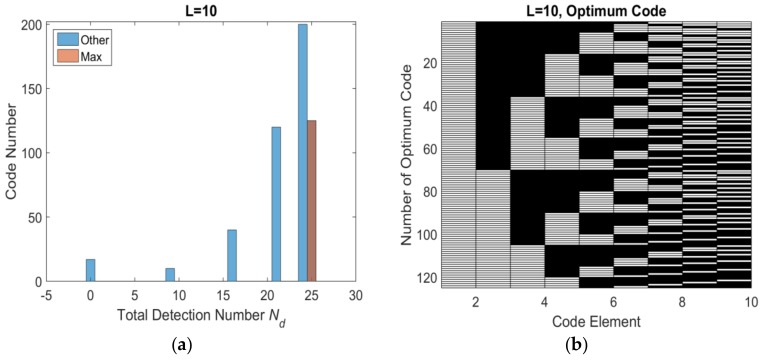
*L* = 10 optimum codes and corresponding *N_d_* (**a**) histogram distribution of *N_d_* (**b**) optimum codes.

Base on the flowchart, the histogram distribution of total number of effective detection windows *N_d_* with different length are shown as Figure 5a, Figure 6a, Figure 7a and Figure 8a. The set of optimum codes corresponding with the maximum *N_d_* are obtained as Figure 5b, Figure 6b, Figure 7b and Figure 8b. When *L* = 7, the maximum of total detection window number *N_d_* is 12 shown in Figure 5, and the corresponding number of optimum codes is 35. The minimum of total detection window number *N_d_* is zero and the corresponding number of invalid codes is 1. By contrast, the maximum of *N_d_* with *L* = 8 is 16 which is larger than *N_d_* with *L* = 7. However, the corresponding number of optimum codes with *L* = 8 is only 32 which is less than that with *L* = 7. Moreover, the number of invalid codes is 8 which is much larger than that with *L* = 7. Although, the code length *L* increases by 1 from *L* = 7 to *L* = 8 and from an odd number to an adjacent and larger even number, the number of optimum codes not only increased but also decreased. The similar result is indicated in *L* = 9 and *L* = 10 shown in Figure 6a and Figure 8a. Hence, the encoding efficiency is highly affected by the code odevity and analyzed in what follows to determine the impact of code length parity on encoding efficiency.

The main calculation procedure is code initialization in step 1, right shift operation in step 2 and exclusive OR operation in step 3. The total number of *L* bits code is 2^(*L*−1)^, the right shift number is *L −* 1 and the exclusive OR operation number is *L −* 1. Hence, the computational complexity of the encoding method proposed in this paper is *O* (2*^N^·N*^2^) and *N* is equal to *L* − 1. As for the spaceborne SAR, the calculation resource is limited. Hence, the transmitting encoding method is designed as an off-line algorithm to save the on-line calculation resource. Firstly, the optimum codes are obtained in the ground center with great calculation resources. Then, the optimum code is switched into a decimal number because each binary code is corresponding with a decimal number. The decimal number is stored in the spaceborne SAR. Finally, when the code length is determined by Equation (13), a random optimum code with corresponding length is selected from the decimal number.

### 3.2. Encoding Efficiency Analysis

As mentioned previously, the total number of codes that can be generated from *L* − 1 bits is 2^(*L*−1)^. Supposing the number of optimum codes is *N_best_*, the ratio of *N_best_* and the total number of codes is defined as encoding factor indicated as follows:

(17) $$G_{b}=\frac{N_{best}}{2^{L-1}}$$

The *N_best_* and maximum of *N_d_* of different even and odd code length *L* are shown in Table 1. It is found that the *N_d_* is increasing with code length *L* regardless the odevity of *L*. However, the *N_best_* of odd code length is larger than that of adjacent even code length.

**Figure 9:**
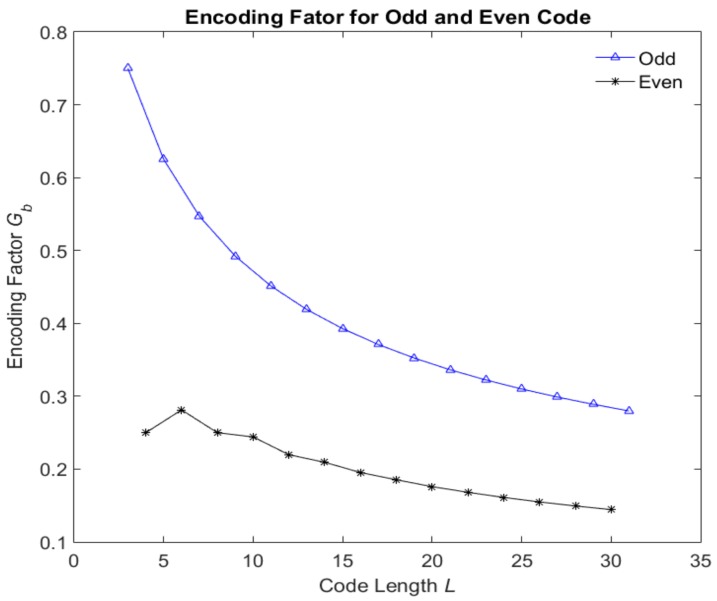
Encoding factors of odd and even codes.

The encoding factors of even and odd codes are shown in Figure 9. It can clearly be seen that the encoding factors of the odd code length are greater than those of the even code length. The encoding factor decreased with the code length. Hence, the optimum codes with even code length are preferred in the beyond-PRT RDJ detection.

### 3.3. Detection Threshold

The fundamental prerequisite for the RDJ and the beyond-PRT RDJ is that the jamming signal must have enough power to cover or blur the protected target. Accordingly, the energy level of the false target formed by jammer is similar to or smaller than that of the protected target in scene.

After the transmitting pulse encoding as Figure 2 shown, there should not have legitimate target echo pulses and theoretically only have range ambiguity signal in the receiving window corresponding to code element 0. Moreover, the power of the range ambiguity signal is much lower than that of the main lobe power of 20 dB.

The energy of the receiving signal corresponded to code element 1 is utilized as detection threshold. The detection threshold *A_th_* is indicated as follows:

(18) $$A_{th}=\frac{\sum_{T\left(i\right)\in 1}\left\|Y_{i}\right\|}{L}$$

where *Y_i_* represents the echoes in the *i*-th receiving window corresponding the *i*-th code element in Equation (10). Hence, the detection threshold is the average energy of echoes from the receiving windows which are corresponding with code element 1 in the total code length. If the receiving signal in the effective detection window is beyond the detection threshold, the beyond-PRT RDJ has entered the receiver. Otherwise, there is no beyond-PRT RDJ.

## 4. Simulation

It is supposed that the satellite is in the nearest Earth orbit with the simulation parameters given in Table 2. The target *P* is located at the middle point of the scene with coordinates (6336.1, 246.8, 34.7) km and the jammer *J* is at coordinates (6364.4, 287.3, 40.4) km, specified in geocentric coordinate system (GCS). The slant ranges from the middle point and the jammer to the satellite, *R_P_* and *R_J_*, are 728.8 km and 745.4 km respectively.

**Figure 10:**
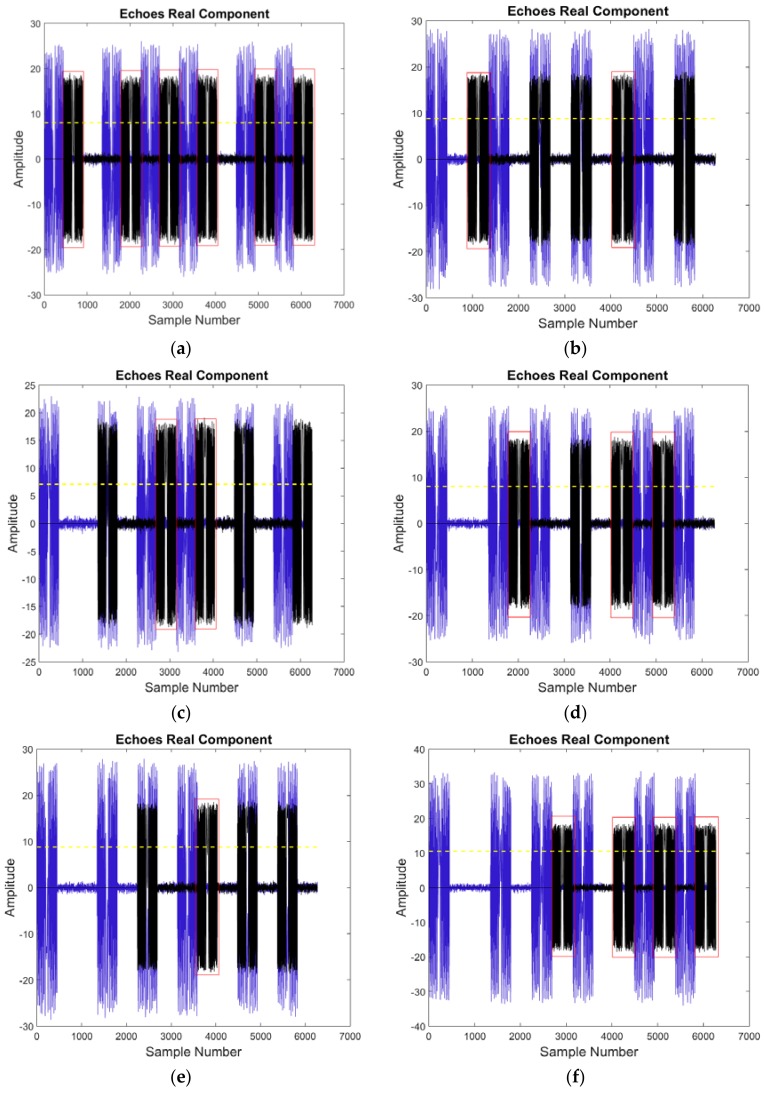
Simulation of beyond-PRT RDJ detection in one dimensional echo, for various time delays of (**a**) 1 PRT; (**b**) 2 PRTs, (**c**) 3 PRTs, (**d**) 4 PRTs, (**e**) 5 PRTs, (**f**) 6 PRTs.

In a scenario, the jammer forms a false target to cover conceal the protected target at the middle point. The maximum effective range of the jammer is taken to be 1200 km. The sub-satellite range is 600 km. Hence, the code length *L* is determined to be 7 using (13) and the maximum time delay for the beyond-PRT RDJ is 6 PRTs. When *L* = 7, the number of optimum codes is 35, as shown in Figure 5. The transmitting code T is [1001010], which is selected from the set of optimum codes and adopted in the simulation. The one dimensional echo signals for each possible time delay, from 1 PRT to 6 PRTs, are shown in Figure 10. The beyond-PRT RDJ signals are included and indicated by the black lines and the target echo signals are indicated by the blue lines. The dashed yellow line indicates the detection threshold and the red rectangles represent the effective detection windows. The time delay of the beyond-PRT RDJ is determined by the relative location of the jammer *J* and protected target *P*, which is from one PRT to six PRTs. As Figure 10 shows, the number of effective detection windows is affected by the time delay of the beyond-PRT RDJ signal. Moreover, the effective detection windows exist for each time delay which means the selected optimum code *T* can continuously detect the beyond-PRT RDJ regardless of the relative location of the jammer *J* and protected target *P*. However, the number of effective detection window is different from the time delay of beyond-PRT RDJ. The least effective detection number is 1 when the time delay is 5 PRTs while the most effective detection number is 6 when the time delay is 1 PRT.

**Figure 11:**
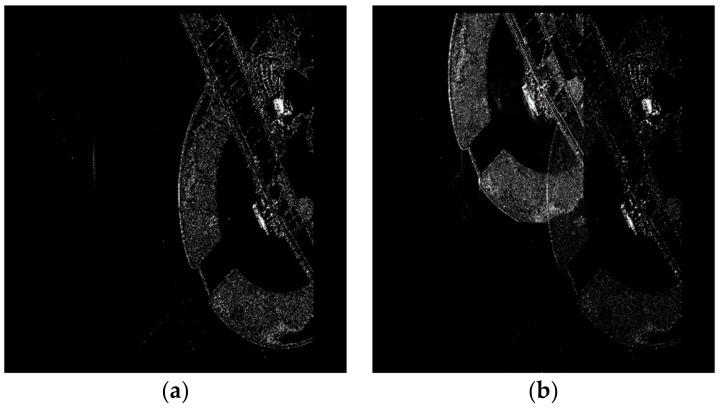
A scene target image, (**a**) target image without RDJ; (**b**) target image with RDJ.

**Figure 12:**
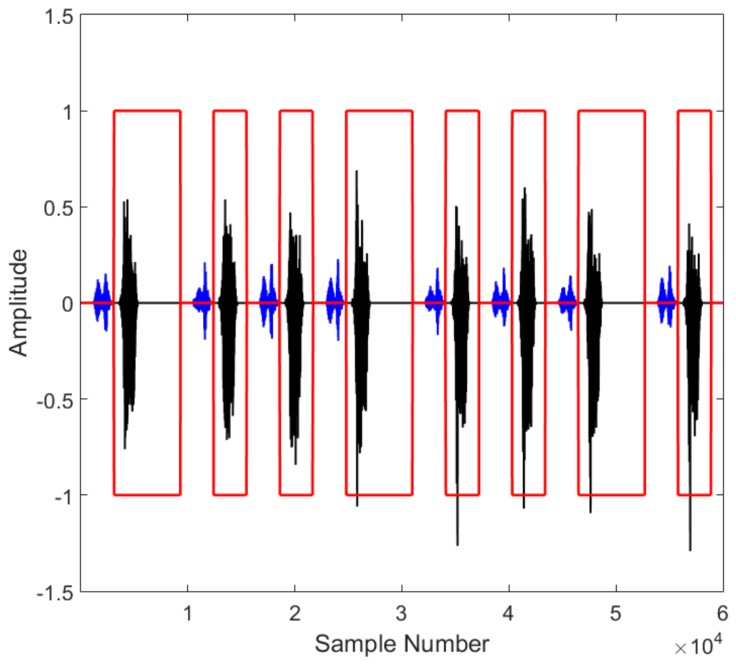
Simulation of beyond-PRT RDJ detection in one dimensional echo of a scene target.

In the same simulation parameters, we simulate a scene target image shown in Figure 11a. In a scenario, the time delay of beyond-PRT RDJ is 1 PRT and the jammer forms a false scene target with 5 dB jamming gain. The back scattering characteristics of the scene target is utilized as the false scene. Hence, a lifelike scene target is imported in the SAR image shown in Figure 11b. The image with beyond-PRT RDJ has negative influence on imaging interpretation. The transmitting code *T* is also utilized to detect the beyond-PRT RDJ in echoes time domain. The simulation result is shown in Figure 12. Echoes in 20 PRTs with transmitting code *T* is arranged in one dimension. The blue line shows the target echoes and the dark line shows the beyond-PRT RDJ signal.

As shown in Figure 12, the beyond-PRT RDJ is effectively detected in the effective detection window, the red rectangle, since the jamming signal energy is usually analogous to the target signal. Hence, the simulation of both point target and scene target has validated the transmitting pulse encoding method in beyond-PRT RDJ detection in spaceborne SAR.

## 5. Conclusions

In this paper, the transmitting pulse encoding method is proposed to detect the beyond-PRT RDJ within spaceborne SAR. The jamming signal can be detected as target echoes are received in time-domain without the need for any transforms and the detection area is controllable through the code length. Based on the encoding method, optimum codes are produced and selected with the ability to continuously detect the beyond-PRT RDJ regardless of the relative location of the jammer *J* and with the most effective detection window. Moreover, the encoding efficiencies of the odd and even codes are analyzed and it is found that the encoding factors of adjacent odd codes is twice that of the adjacent even codes. The simulation results validate the proposed transmitting pulse encoding method. In the future, we will investigate the RDJ detection method in airborne SAR by using transmitting pulse and modulation rate encoding to improve the electromagnetic environment perceiving capability of SAR.

## Figures and Tables

**Table 1:** Number of optimum codes and Maximum detection window number in different code length.

Odd			Even		
*L*	*N_d_*	*N_best_*	*L*	*N_d_*	*N_best_*
5	6	10	6	9	9
7	12	35	8	16	32
9	20	126	10	25	125
11	30	462	12	36	450
13	42	1716	14	49	1715
15	56	6435	16	64	6400
17	72	24,310	18	81	24,300
19	90	92,378	20	100	92,250

**Table 2:** Simulation parameters.

Parameter	Value
Orbit Height	680 km
Eccentricity	0.01
Orbit Inclination	98
Pitch Angle	20°
Incident Angle	23.8°
PRF	2000 Hz
Band Width	50 MHz
